# Anti-Wrinkle and Anti-Inflammatory Effects of Active Garlic Components and the Inhibition of MMPs *via* NF-κB Signaling

**DOI:** 10.1371/journal.pone.0073877

**Published:** 2013-09-16

**Authors:** So Ra Kim, Yu Ri Jung, Hye Jin An, Dae Hyun Kim, Eun Ji Jang, Yeon Ja Choi, Kyoung Mi Moon, Min Hi Park, Chan Hum Park, Ki Wung Chung, Ha Ram Bae, Yung Whan Choi, Nam Deuk Kim, Hae Young Chung

**Affiliations:** 1 Molecular Inflammation Research Center for Aging Intervention (MRCA), College of Pharmacy, Pusan National University, Busan, Republic of Korea; 2 Department of Horticultural Bioscience, Pusan National University, Miryang, Republic of Korea; Johns Hopkins School of Medicine, United States of America

## Abstract

Skin aging is a multisystem degenerative process caused by several factors, such as, UV irradiation, stress, and smoke. Furthermore, wrinkle formation is a striking feature of photoaging and is associated with oxidative stress and inflammatory response. In the present study, we investigated whether caffeic acid, *S*-allyl cysteine, and uracil, which were isolated from garlic, modulate UVB-induced wrinkle formation and effect the expression of matrix-metalloproteinase (MMP) and NF-κB signaling. The results obtained showed that all three compounds significantly inhibited the degradation of type І procollagen and the expressions of MMPs *in vivo* and attenuated the histological collagen fiber disorder and oxidative stress *in vivo*. Furthermore, caffeic acid and *S*-allyl cysteine were found to decrease oxidative stress and inflammation by modulating the activities of NF-κB and AP-1, and uracil exhibited an indirect anti-oxidant effect by suppressing cyclooxygenase-2 (COX-2) and inducible nitric oxide synthase (iNOS) expressions levels and downregulating transcriptional factors. These results suggest that the anti-wrinkle effects of caffeic acid, *S*-allyl cysteine, and uracil are due to anti-oxidant and/or anti-inflammatory effects. Summarizing, caffeic acid, *S*-allyl cysteine, and uracil inhibited UVB-induced wrinkle formation by modulating MMP *via* NF-κB signaling**.**

## Introduction

Skin aging is a multisystem degenerative process that involves the skin and the skin support system [1]. Skin aging may be caused by several factors, such as, UV irradiation, stress, or smoking. Acute exposure of human skin to UV irradiation causes sunburn, inflammation, immune suppression, and dermal connective tissue damage [2], and chronic exposure to UV over many years disrupts the normal skin architecture and ultimately causes photoaging and even skin cancer [3]. Wrinkle formation is a striking feature of photoaged skin and is caused by the degradation of collagen fibrils and gelatin fibers.

The UV spectrum is classified by wavelength as UVA (320-400 nm), UVB (280-320 nm), and UVC (200-280 nm). UVA and UVB reach the earth’s surface but UVC is filtered out by the ozone layer [4]. UVA and to a lesser extent UVB are responsible for sunlight induced skin cancer [5]. UVA accounts for 90-99% of the UV energy that reaches the earth’s surface and UVB contributes the other 1-10% [6]. However, UVB has been reported to be 1,000-10,000 times more carcinogenic than UVA [7]. Furthermore, over several years, systematic ozone depletion has been occurred due to human activities, predominantly because of emissions of halogen-containing compounds, and this has increased UVB levels on the surface of the earth and consequently the amount of UVB absorbed by skin.

UV increased release of pro-inflammatory mediators from a variety of skin cells, resulting in MMP activation [8]. However, inflammation activated various matrix-degrading MMP, which leads to abnormal matrix degradation and accumulation of non-functional matrix components [9]. Thus, MMP is used for major marker of UVB-induced wrinkle as well as skin-inflammation. In addition, reactions to UV-induced inflammation in skin were enhanced in aging skin, such as deep wrinkles and thickening of the dermis and epidermis [10,11].

UV damages *de novo* type І collagen synthesis, and UV-induced AP-1 downregulates type І collagen, the most abundant protein in skin connective tissue, which also upregulates the transcriptions of the COL1A1 and COL1A2 genes [8]. This process, called collagen degradation, results in the expressions of MMPs, which are responsible for wrinkle formation in photodamaged skin [12].

AP-1 tightly regulates the transcriptions of several MMPs (matrix-metalloproteinase). In particular, MMP1 (interstitial collagenase or collagenase 1), MMP9 (gelatinase B), and MMP3 (stromelysin 1) are upregulated by AP-1. MMP1 initiates the degradations of collagens type I and III, and MMP9 further degrades collagen fragments generated by collagenases. MMP3 not only degrades basement membrane type IV collagen but also activates proMMP1. UV-induced damage to skin connective tissue requires MMP induction, and collectively, MMP1, MMP3, and MMP9 completely degrade mature collagen in the skin. Accordingly, it is known that UV irradiation causes extracellular matrix degradation *via* the induction of transcription factor AP-1 and subsequent increases in the productions of MMPs in human skin [13].

Garlic has several effects, that is, it acts as an antioxidant, inhibits NF-κB activation, and protects against UV-induced immunity suppression [14]. Caffeic acid (CA) is found in garlic, fruits, and coffee contains both phenolic and acrylic functional groups. CA is a well-known pharmacological antioxidant with antimutagenic activities and anti-inflammatory and immunomodulatory effects. Interestingly, some studies have also shown that carcinogenesis is inhibited by CA. In this study, CA is used for positive control because that is well-known for possible anti-wrinkle agent. CA suppressed UVB-induced photoaging by inhibiting MMPs and elevating type І procollagen production through ROS scavenging and down-regulation of MAP kinases pathway [15]. Thus, we investigated anti-wrinkle effect of active garlic compounds comparing with CA *in vivo*.

**Figure 1:**
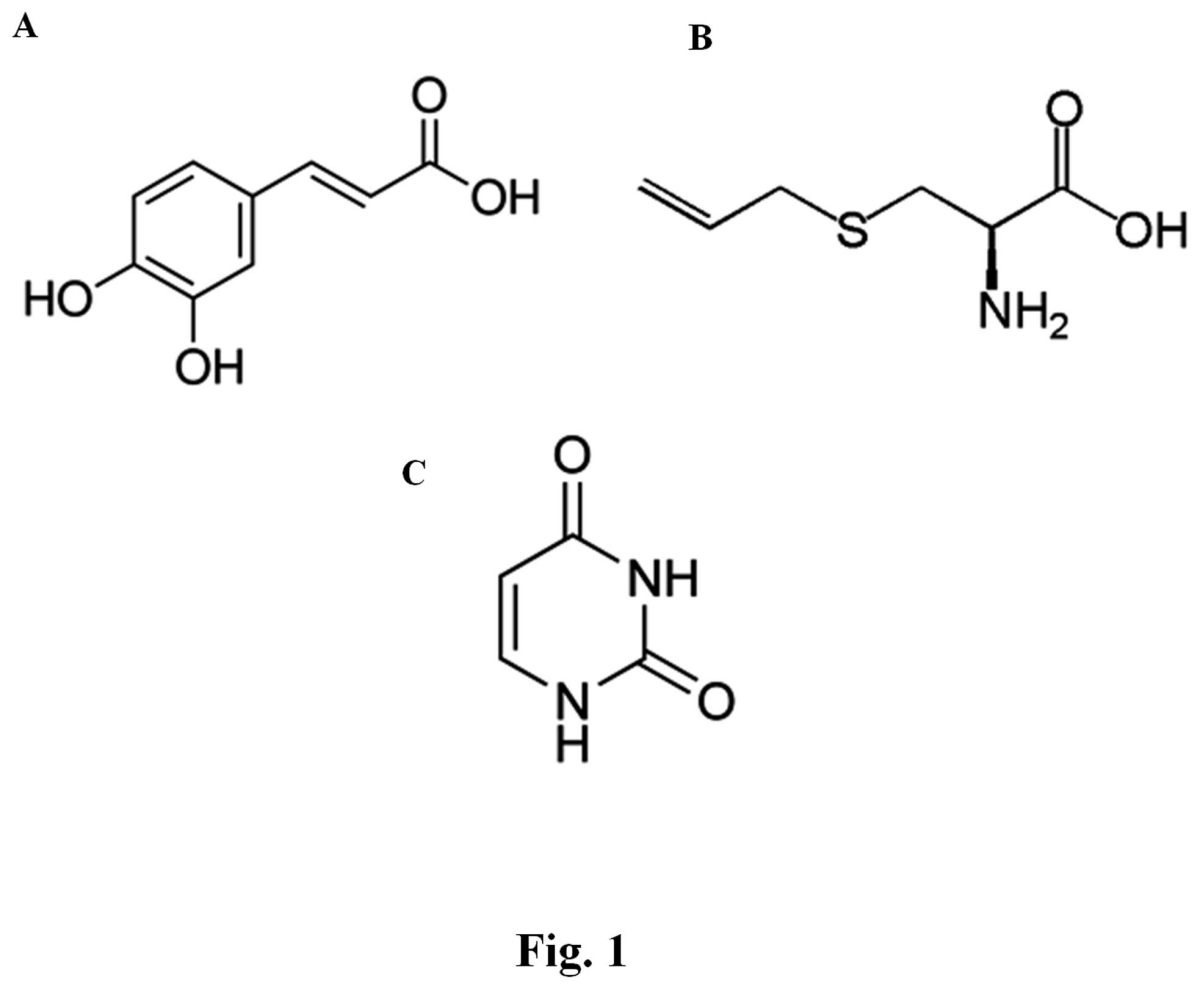
Structures of active the three garlic compounds. (A) caffeic acid, (B) *S*-allyl cysteine, (C) uracil.

Given that S-allyl cysteine (SAC) is the most abundant compound in AGE (aged garlic extract), the present paper draws attention to the physicochemical characteristics, toxicity, pharmacokinetics, tissue distribution, and metabolism of this compound and to the different antioxidant mechanisms underlying its protective actions in different experimental models of toxicity [16]. Uracil is also present in garlic, and has a well-known anticancer effect and increases cell viability. Despite its widely known biological activities, the molecular mechanisms by which active garlic compounds, such as, CA, SAC, and uracil (Figure 1), affect intracellular activities in skin have not been explored.

The purpose of the present study was to investigate the mechanisms responsible for the effect of the active components of garlic on UVB-induced wrinkling. Accordingly, we examined the inhibitory effects of active garlic compounds on UVB-induced wrinkle formation, collagen fiber destruction, and on the expressions of MMPs *in vivo* using a HR1 hairless mouse model.

It is hoped that the evidence gathered during this study provides significant new information on anti-wrinkle effects and molecular insight of the activities of active garlic compounds during UVB-induced photoaging. 

## Results

### 2.1: Histological changes in UVB-irradiated skin by active garlic compounds

**Figure 2:**
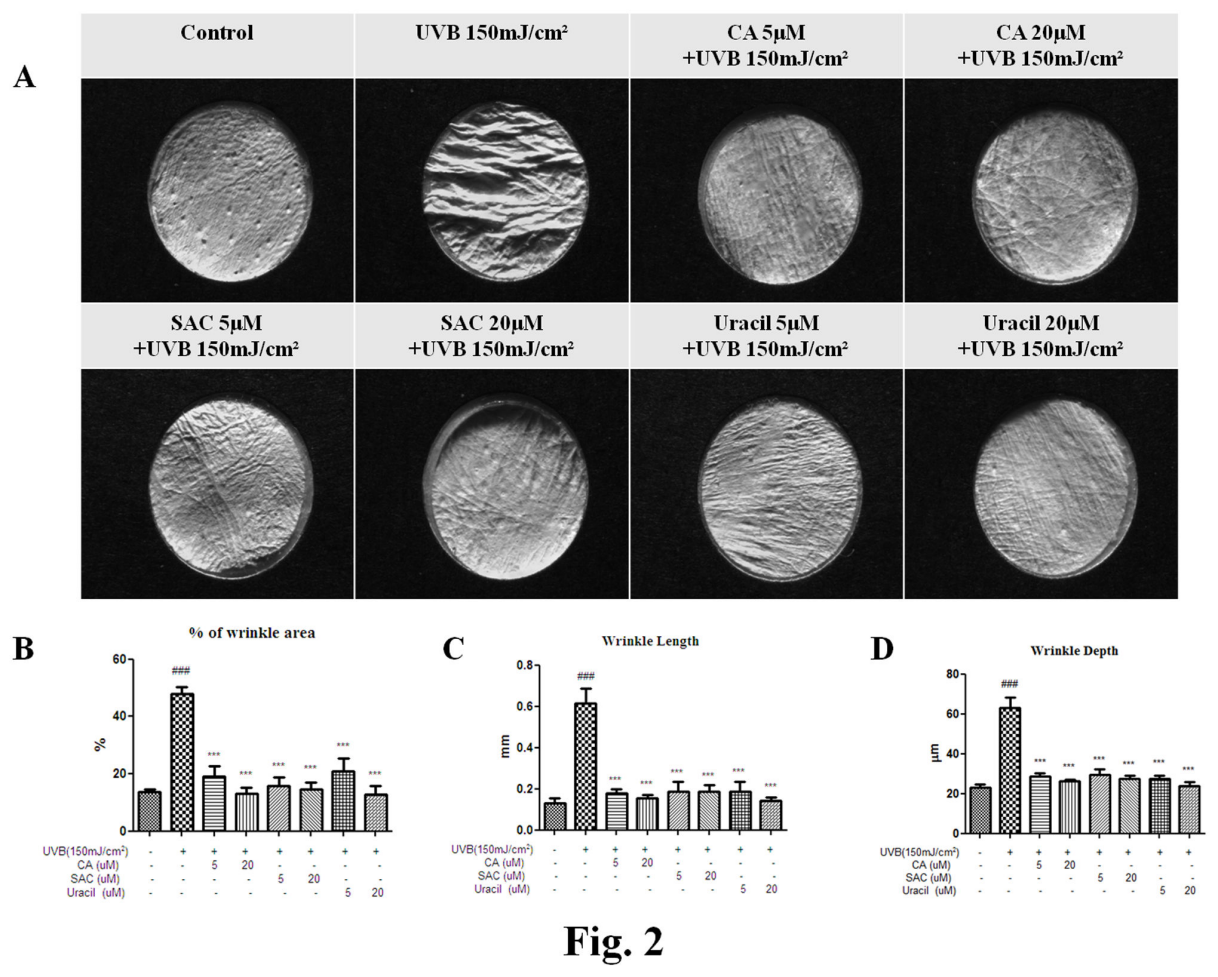
Active compounds from garlic decreased wrinkle depth in hairless mice. (A) Representative images of skin replicas showing that the histological formation of wrinkles was reduced by the three garlic compounds as compared with UVB-treated animals, (B) wrinkle area (%), (C) wrinkle length, and (D) wrinkle depth.

Histologic examinations of skin samples from control mice right backs (Figure 2; x200) and skin samples from UV irradiated right backs showed a thinning of the deep dermis. In this study, CA is used as positive control because that is well-known for possible anti-wrinkle agent. As shown in Figure 2, dermis treated with CA, SAC, or uracil showed significant wrinkle recovery as compared with dermis treated with UVB.

### 2.2: Histopathological changes of collagen by active compounds from garlic in hairless mice skin tissue

**Figure 3:**
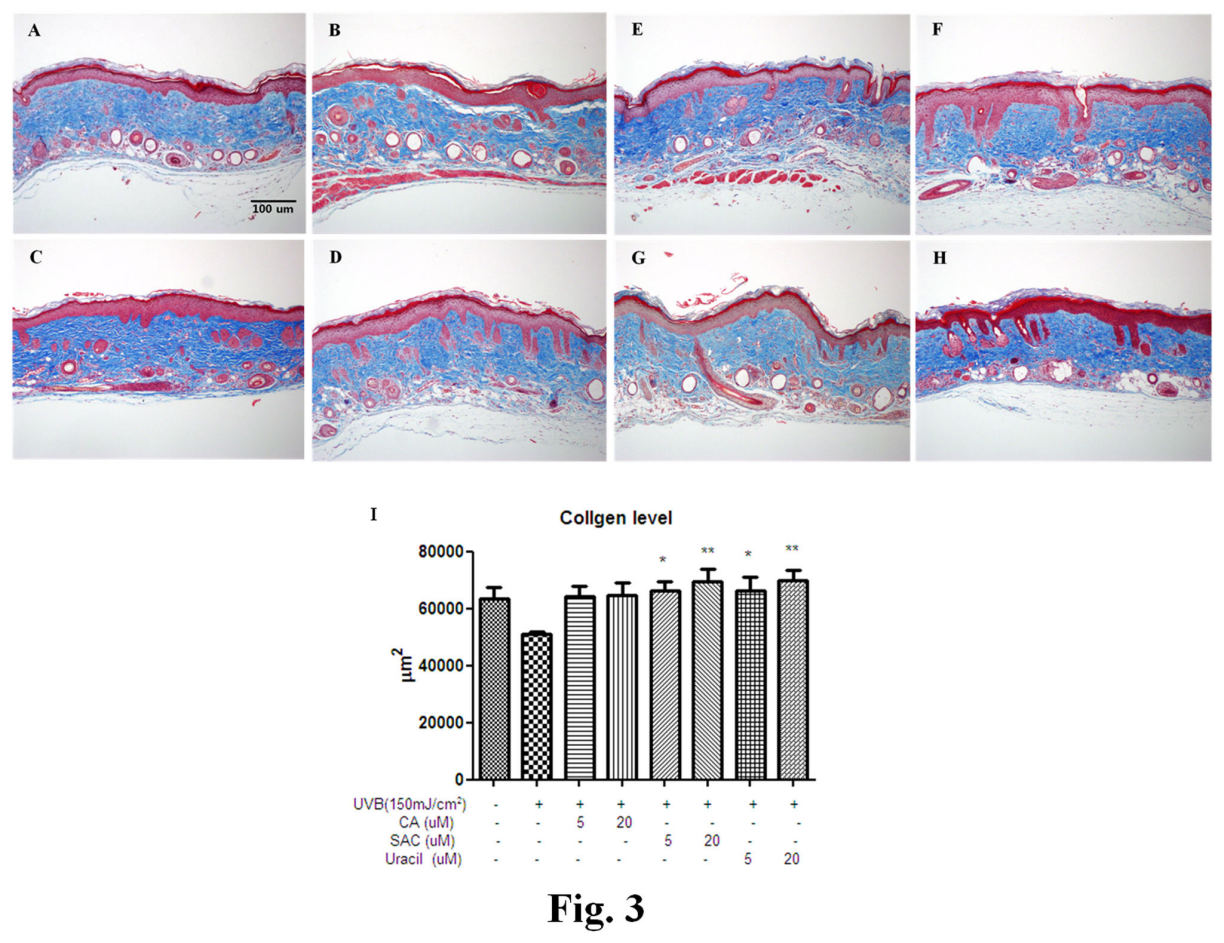
Histopathological analyses of collagen in hairless mouse skin tissues. Skin samples were processed and stained with Masson’s Trichrome as described in Materials and Methods. Collagen staining appears blue (original magnification × 200). (A) control, (B) UVB 150 mJ/cm^2^, (C) pretreated with CA 5µM 2hr before UVB exposure, (D) pretreated with CA 20µM 2hr before UVB exposure, (E) pretreated with SAC 5µM 2hr before UVB exposure, (F) pretreated with SAC 20µM 2hr before UVB exposure, (G) pretreated with uracil 5µM 2hr before UVB exposure, (H) pretreated with uracil 20µM 2hr before UVB exposure; CA, caffeic acid; SAC, *S*-allyl cysteine; UVB, ultraviolet B.

The histological appearances of UVB-irradiated skins treated with active garlic compounds were determined by Masson’s trichrome staining. As shown in Figure 3, UVB-irradiation caused collagen fiber destruction, but CA, SAC, and uracil pretreatments ameliorated this effect (Figure 3B).

### 2.3: Upregulation of type І procollagen production by active compounds from garlic

Type I collagen is the most abundant protein in skin connective tissue. Newly synthesized type I procollagen is secreted into the dermal extracellular space, and has an anti-wrinkle effect in UVB-irradiated skin.

**Figure 4:**
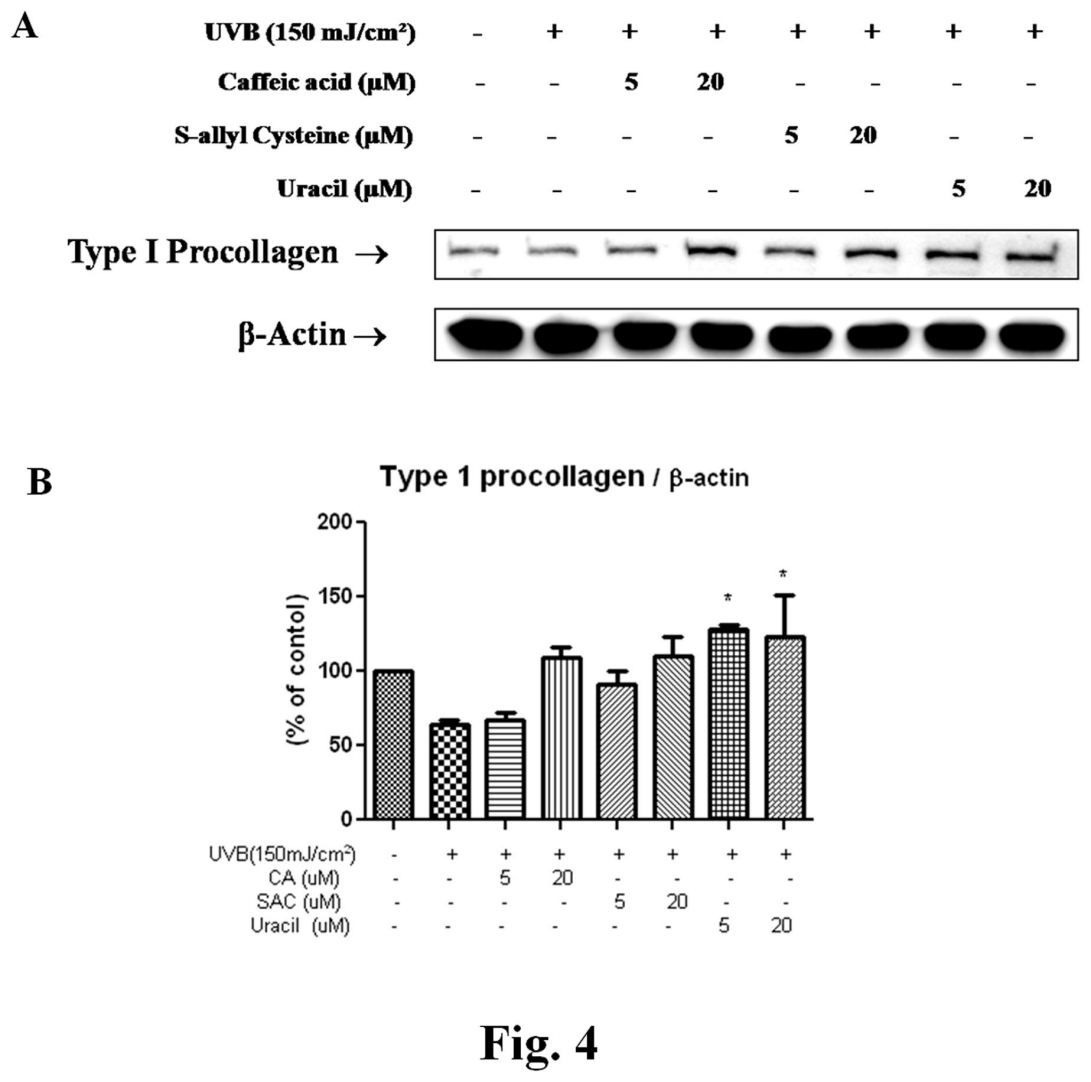
Effects of the three garlic compounds on type І procollagen level by UVB in hairless mice. (A) Western blotting was performed to detect cytoplasmic extracts of UVB-irradiated hairless mouse skin. (B) Blots were quantified by densitometry as percentages of control. One-factor ANOVA were used to determine significance: ^*^p<0.05 vs. control. CA, caffeic acid; SAC, *S*-allyl cysteine, UVB, ultraviolet B.

As shown in Figure 4A, levels of type I procollagen suppressed by UVB (at 150 mJ/cm^2^) were restored by pretreatment with CA, SAC, or uracil.

### 2.4: Modulation of UVB-induced MMPs by the active compounds from garlic

MMP induction is, in part, responsible for UV-induced damage to skin connective tissues. MMPs have the ability to degrade mature fibrillar collagen completely. In addition to impairing collagen synthesis, increased levels of several MMP family members, including MMP13 (collagenase 3), MMP3 (stromelysin 1), MMP9 (gelatinase B), and MMP12 (macrophage elastase) occurs in chronologically aged skin [17]. These observations are consistent with induction of transcription factors NF-κB and AP-1 by UVB irradiation.

**Figure 5:**
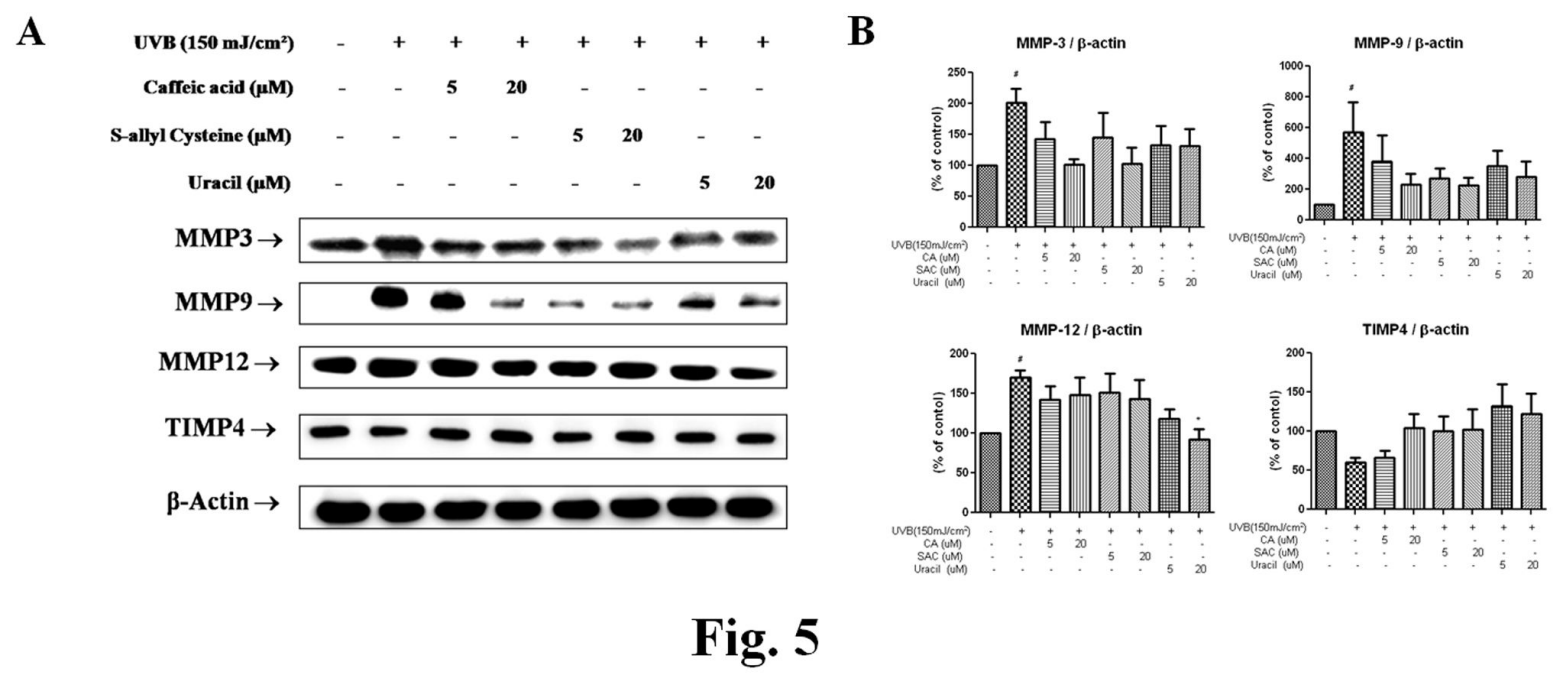
Modulation of MMP expression in mice by the three garlic compounds. (A) Western blot analysis was performed to detect MMP3, MMP9, MMP12, and TIMP4 protein levels in cytosolic extract of the skins of from hairless mice. (B) Blots were quantified by densitometry as percentages of the control. One-factor ANOVA was used to determine significance: ^#^ p<0.05 vs. control, *p<0.05 vs. the UVB-irradiated group; CA, caffeic acid; MMP, matrix metalloproteinase; SAC, *S*-allyl cysteine; TIMP4, tissue inhibitor of metalloprotease4; UVB, ultraviolet B.

As shown in Figure 5A, levels of MMP3 and MMP9 protein were elevated in UVB (150 mJ/cm^2^)-treated groups versus the control group but pretreatment with CA, SAC, or uracil suppressed these levels. MMP12 level reductions by UVB were also prevented by pretreatment with uracil, and TIMP4, the unfamiliar inhibitor of matrix metalloproteinase, level reductions by 150 mJ/cm^2^ of UVB were prevented by pretreatment with any of the three active compounds. These results suggest that the three active compounds protect skin connective tissue from UVB via the suppression of MMPs and TIMP4 degradation.

### 2.5: Effects of active compounds from garlic on MMPs-related transcription factor during photoaging

**Figure 6:**
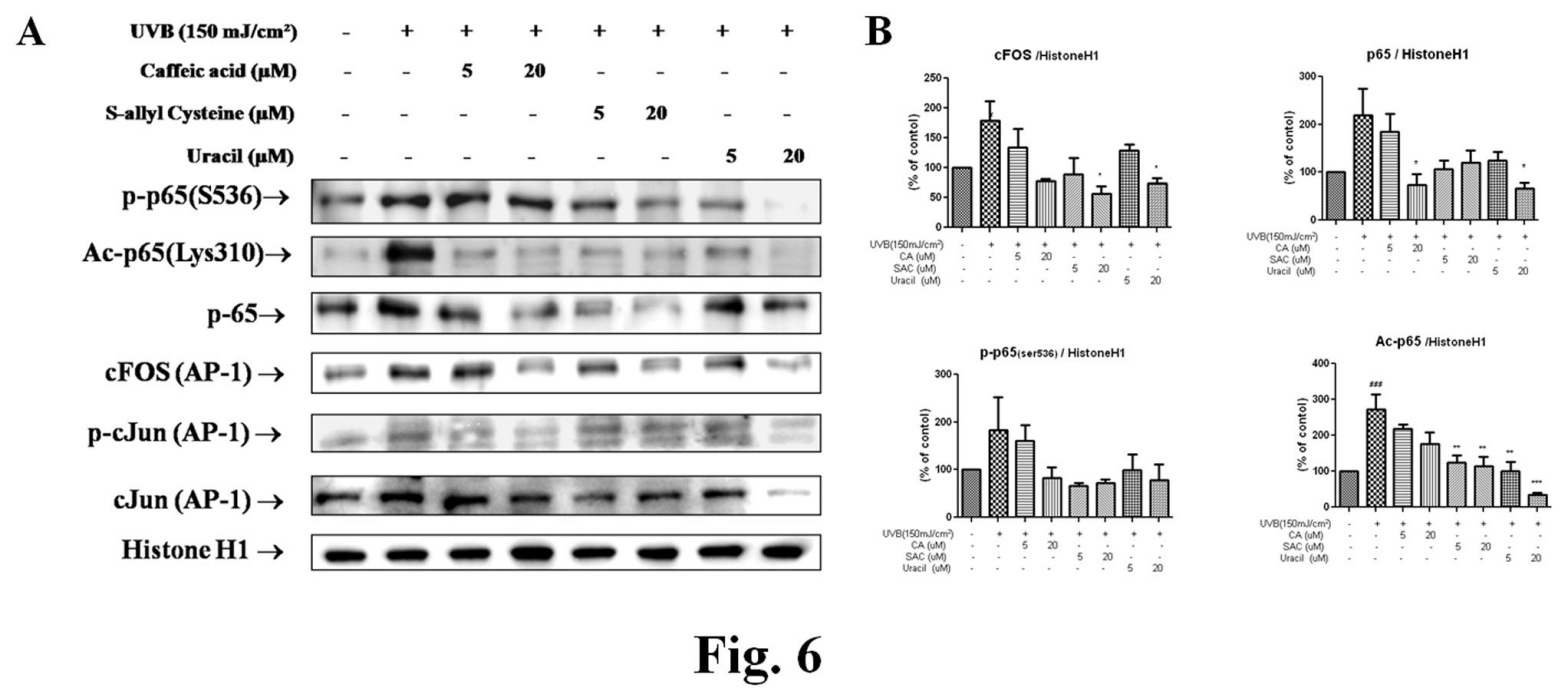
Changes in the activities of nuclear NF-κB and AP-1 caused by three compounds in UVB-irradiated hairless mice. (A) Western blotting was performed to determine nuclear p50, p-p65, Ac-p65, p65, cFOS, and p-cJun protein levels in nuclear extracts of the skins of UVB-irradiated hairless mice. The protein levels of NF-κB family members and of AP-1 component increased after UVB irradiation, but these increases were reduced by all three compounds. (B) Blots were quantified by densitometry as percentages of the control. One-factor ANOVA was used to determine significance: ^# # #^ p < 0.001 vs. control, ^*^p<0.05, **p < 0.01 and ***p < 0.001 vs. UVB-irradiated group respectively. AP-1, activator protein 1; UVB, ultraviolet B.

To assess the changes in NF-κB and AP-1 transcription factors by active compounds in UVB-treated mice skin, Western blot analysis was performed. The protein levels of NF-κB family, including p-p65, p65, and acetyl-p65, were increased by UVB but decreased by the three garlic compounds. Likewise, these compounds also significantly decreased cFOS and p-cJun (components of AP-1) levels (Figure 6).

### 2.6: Active compounds from garlic inhibited the expressions of NF-κB-dependant genes

**Figure 7:**
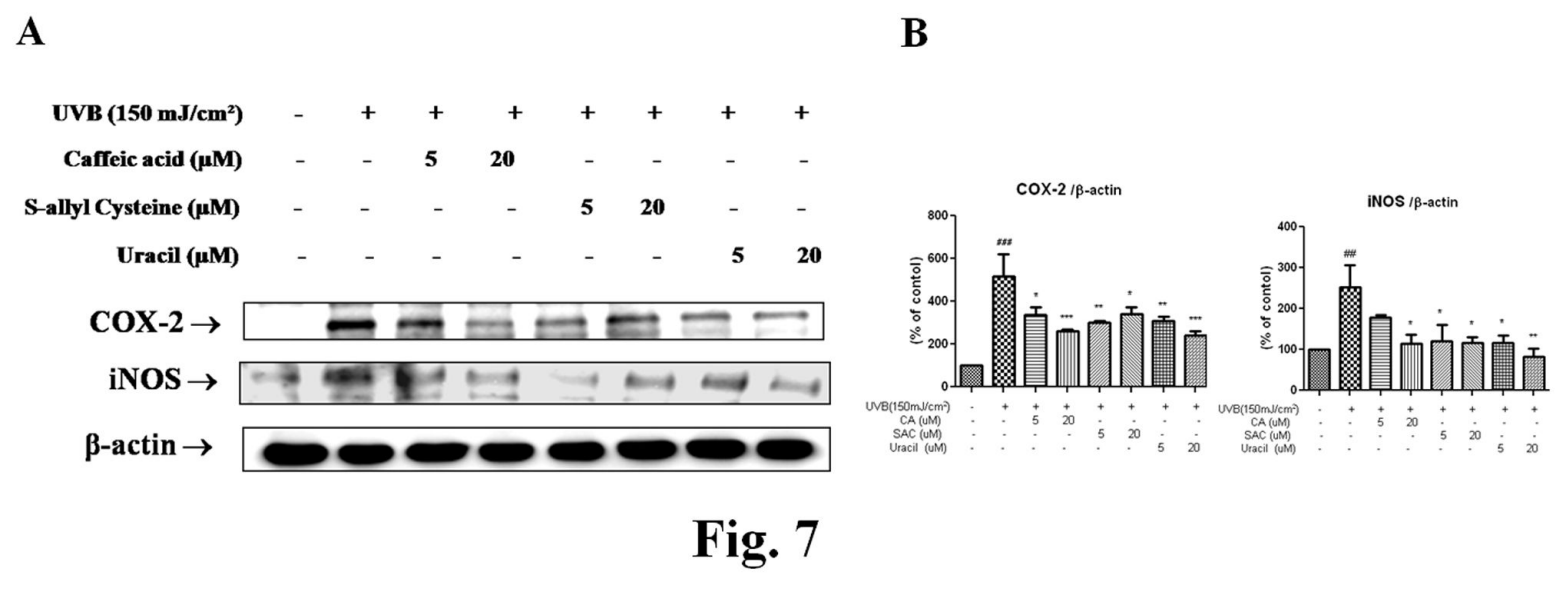
Inhibition of the expressions of NF-κB-dependant genes by the three garlic compounds. (A) To determine the effect of UVB on the expressions of NF-κB dependent genes, we examined the expressions of COX-2 and iNOS *in* *vivo*. (B) Blots were quantified by densitometry as percentages of the control. One-factor ANOVA was used to determine significance: ^# # #^ p < 0.001 vs. control, ^*^p<0.05, **p < 0.01 and ***p < 0.001 vs. UVB-irradiated group.

To determine the effect of UVB on the expressions of NF-κB dependent genes, we examined the expressions of COX-2 and iNOS. These genes are known to be associated with inflammation and to possess an NF-κB binding site in their promoter regions. As shown in Figure 7, COX-2, and iNOS protein levels were increased by UVB, but pretreatments with CA, SAC, and uracil decreased these increases (Figure 6). These results suggest that CA, SAC, and uracil modulate NF-κB activation and the expressions of NF-κB-dependant genes.

### 2.7: Changes in NF-κB signaling pathway by active compounds from garlic

**Figure 8:**
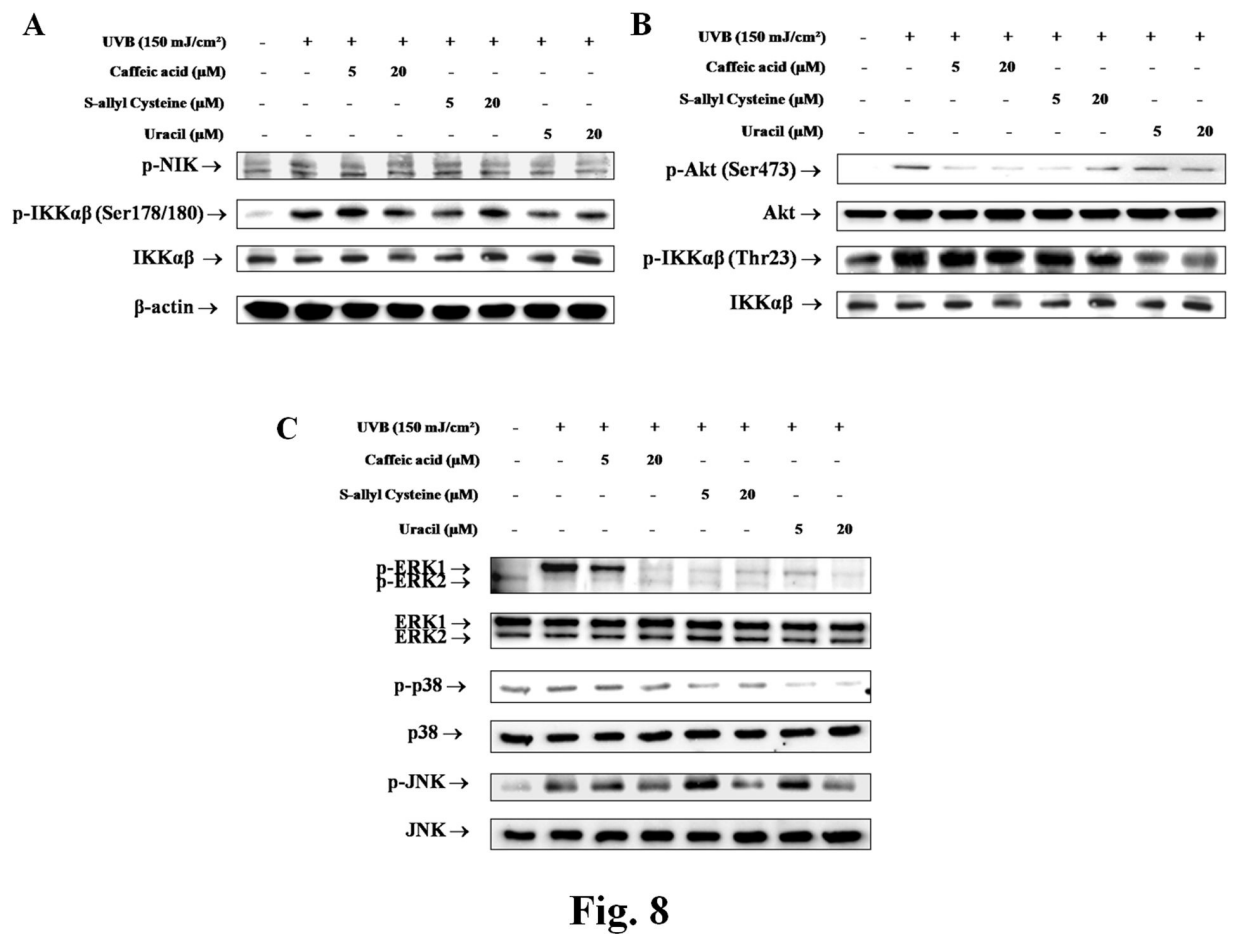
Modulation of the NF-κB signaling pathway the three compounds. Western blotting was performed on cytoplasmic extracts from UVB-irradiated hairless mice. (A) (B) Phosphorylations of NIK, Akt and IKK were quantified as p-NIK, p-Akt, and p-IKKα/β, respectively, and MAPK phosphorylation was detected using antibodies for p-ERK, p-p38 and p-JNK. ERK (extracellular regulated signal kinase) in UVB-irradiated hairless mouse skin, (C) Western blotting was performed to quantify p-ERK, p-p38, and p-JNK protein levels in UVB-irradiated hairless mice skin; JNK, c-Jun N-terminal kinases; NF-κB, nuclear factor-kappa B; NIK, NF-κB-inducing kinase; IKK, IκB kinase; COX-2, cyclooxygenase-2; UVB, ultraviolet B.

As shown in Figure 8, Akt activation was significantly increased by UVB, but pretreatment with CA, SAC, and uracil reduced these activations. Levels of phospho-IKKαβ (Ser176/180) and phospho-IKKαβ (Tyr23) were also considerably decreased, and phospho-NIK, the upstream gene of phospho-IKKαβ (Ser176/180) in hairless mice, was strongly downregulated all three compounds (Figure 8A and 8B). In addition, MAPK phosphorylation was detected using antibodies for p-ERK1/2, p-JNK, and p-p38. The protein levels of all three were increased by UVB skin, but CA, SAC, and uracil pretreatments decreased these increases (Figure 8C).

### 2.8: Inhibitory effects of the three compounds on UVB-induced oxidative stress

Oxidative stress is a primary factor in the photoaging process. UVB increases levels of hydrogen peroxides and other reactive oxygen species in skin and decreases the levels of anti-oxidant enzymes [18]. These features are also observed in chronologically aged human skin. In both cases, increased ROS production alters gene and protein structures and functions and ultimately leads to skin damage. ROS are necessary participants in multiple MAP kinase pathways, and MAPK activation results in the inductions of NF-κB and/or AP-1, which, in turn, upregulate the expressions of MMPs. This cascade provides a mechanism for the increased collagen degradation observed in photoaged skin.

**Figure 9:**
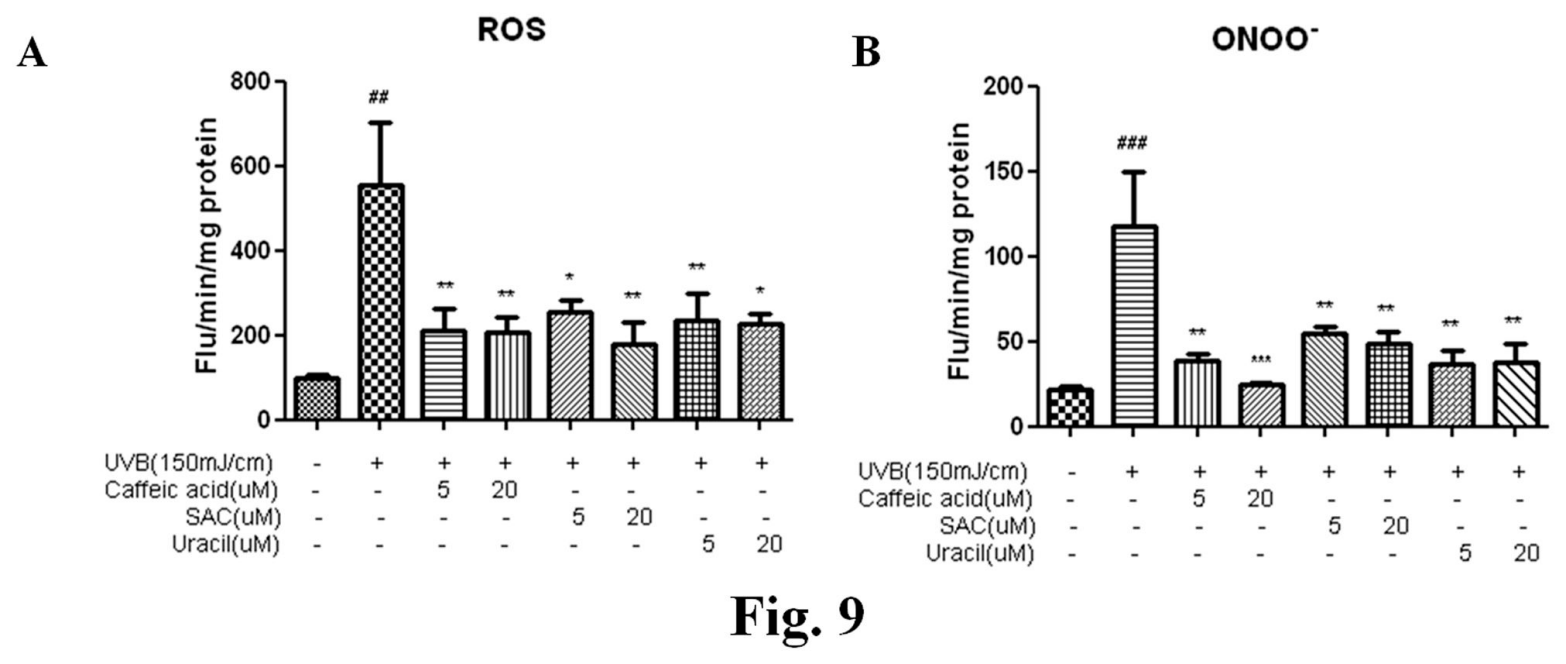
Effects of the three compounds on intracellular ROS/ONOO¯ in UVB-irradiated hairless mice. (A) (B) ROS and ONOO**¯** generations by UVB in hairless mouse skins were decreased concentration-dependently by pretreating animals with each of the three compounds. Results are expressed as means±SEs of three determinations. Significance was determined using one-factor ANOVA: ^# # #^ p < 0.001, ^# #^ p < 0.01 vs. control; *p < 0.05, **p < 0.01 and ***p < 0.001 vs. UVB-irradiated group; CA, caffeic acid; COX-2, cyclooxygenase-2; iNOS, inducible nitric oxide synthase; MMP1, matrix metalloproteinase1; MMP3, metalloproteinase3; MMP9, metalloproteinase9; MMP13, matrix metalloproteinase13; ONOO¯, peroxynitrite; ROS, reactive oxygen species ; UVB, ultraviolet B.

To assess UVB-induced oxidative statuses, total ROS levels were measured using a DCFDA probe in skin homogenates. The increased levels of ROS post-UVB irradiation were found to be suppressed by pretreatment with CA, SAC, or uracil in a dose-dependent manner (Figure 9A). Furthermore, ONOO¯ levels were higher in UVB-irradiated group compared to untreated group, showing that CA, SAC, and uracil pretreatments inhibited ONOO¯ generation (Figure 9B).

## Discussion

Photoaging is the result of the superposition of repetitive ultraviolet (UV)-induced damage and intrinsic aging and accounts for most age-associated changes in skin [2]. Photoaging is triggered by receptor-initiated signaling, mitochondrial damage, protein oxidation, and telomere-based DNA damage responses [19]. UV-irradiated skin displays variable epidermal thickness, increased collagen fragmentation, increased levels of matrix-degrading metalloproteinases, dermal elastosis, inflammation, and vessel ectasia [20]. In addition, wrinkle formation is a striking feature of photoaged skin and is caused by the degradation of collagen fibrils and gelatin fibers [21].

This study shows that UVB irradiation increases wrinkle formation and collagen disorder. Although these data clearly demonstrated the anti-wrinkle effect of active garlic compounds *in vivo*.

UVB-induced skin damage has been extensively studied from the perspective s of wrinkle formation and skin inflammation. The most significant cause of photodamage is oxidative stress, which is considered to play a central role in initiating and driving the signaling events that lead to cellular responses to UV exposure. Furthermore, UV irradiation of skin has been reported to increase levels of hydrogen peroxide and ther reactive oxygen species (ROS) [22], and to reduce levels of anti-oxidant enzymes [18]. The anti-oxidant effects of CA and SAC have been previously examined [22]. Furthermore, CA and SAC have been reported to scavenge the superoxide and peroxynitrite anions [23]. Accordingly, these studies and the present study strongly indicate that CA and SAC inhibit oxidative stress. In the present study, it was interesting to find that uracil was able to lower ROS and peroxynitrite levels significantly in UVB-irradiated hairless mice (Figure 9).

Major signaling pathways known to mediate UVB-induced biological responses involve mitogen-activated protein kinases (MAPKs) [24]. MAPKs mediate a wide range of intracellular signaling molecules that are involved in processes, such as, inflammation, cell proliferation, differentiation, and apoptosis. Three types of MAPKs have been characterized, that is, the extracellular signal-regulated kinases (ERKs), the c-Jun N-terminal kinases (JNKs), and p38. The phosphatidylinositol-3 kinase (PI-3K) pathway is another key activator of UVB-induced cellular responses. PI-3K is a major kinase upstream of Akt [25], and the PI-3K pathway regulates various cellular processes, such as, apoptosis, proliferation, and growth, and requires UVB-induced NF-κB activation via the phosphorylation of IKKαβ (Thr23) [26]. However, the molecular mechanism leading to MAPK activation by UV-induced ROS production has not been elucidated. In previous studies, CA and FA were found to protect HaCaT cells against UVA-induced MMP1 activation and ROS formation [27]. Likewise, in the present study, CA decreased UVB-induced MMP expression and ROS formation (Figure 5). Increased ROS levels act to amplify the signal leading to the activations of MAPKs, Akt, and IKKαβ. The findings of the present study are in-line with those of recent present studies (Figure 8).

UVB-induced cellular damage at the DNA and molecular levels initiates the activations of transcription factor pathways, which in turn regulate the expressions of a number of genes, such as, COX-2, MMPs, TNFα, and IL-1. Nuclear factor-κB (NF-κB) and activator protein-1 (AP-1) are two representative transcription factor families. NF-κB regulation can also be influenced by signaling cascades that are involved in the activation of AP-1 [28]. Thus, the activations of specific signaling pathways and their interactions with NF-κB and the dual inhibition of AP-1 seem to offer promising strategies for anti-inflammatory and anti-wrinkle treatments. Thus, we investigated whether transcription factors, such as, NF-κB and AP-1, are influenced by active garlic compounds (Figure 6).

In the present study, the level of nuclear p65 decreased in a dose-dependent manner *in vivo* after treatment with CA, SAC, or uracil. Furthermore, these compounds downregulated cFOS levels, which were significantly increased by UVB (Figure 6). In addition, the modulation of transcription factors seems to regulate the ability of uracil to scavenge ROS *in vivo*.

The increased activities of AP-1 and NF-κB downregulate type I procollagen and TIMP (tissue inhibitor of metalloproteinases) and upregulates MMPs. TIMP4 is the newest member of the mammalian TIMP family and has an expression pattern that differs from those of the other 3 TIMPs. The expressions of these genes are remarkably reduced by UVB irradiation and aging, whereas UVB increases the synthesis of matrix metalloproteinases (MMPs). Each MMP member has a unique function in UVB-irradiated skin. MMP1 is primarily responsible for degradation of ECM [29], and increased expressions of MMP1 and MMP3 (a collagen decomposition enzyme) are believed to cause collagen degradation. In skin, MMP9 is thought to play an important role in the final degradation of fibrillar collagens after initial cleavage by collagenases, such as, MMP1, MMP8, and MMP13. Furthermore, it has been reported that the gelatinase activity of MMP9 cleaves collagens types I, II, and V in the N Terminal NonHelical Telopeptide [30]. Therefore, it is possible that MMP9 plays a much broader role in the remodeling of collagenous ECM than has been previously thought. In addition, MMP12 is the most active MMP against elastin [31]. Recently, the catalytic domain of MMP12 was reported to bind and degrade collagens I and III [32]. In the present study, levels of MMP3, MMP9, and MMP12 were increased by 150 mJ/cm^2^ of UVB and these increases were downregulated by CA, SAC, or uracil pretreatment *in vivo*. In addition, decreases in type I procollagen levels were prevented by pretreatment with CA, SAC, or uracil (Figure 4), which suggests their potential uses as wrinkle suppressants. Furthermore, it is remarkable that uracil effectively inhibited the inductions of MMP gene expressions by UVB and procollagen production, which is reported here for the first time.

**Figure 10:**
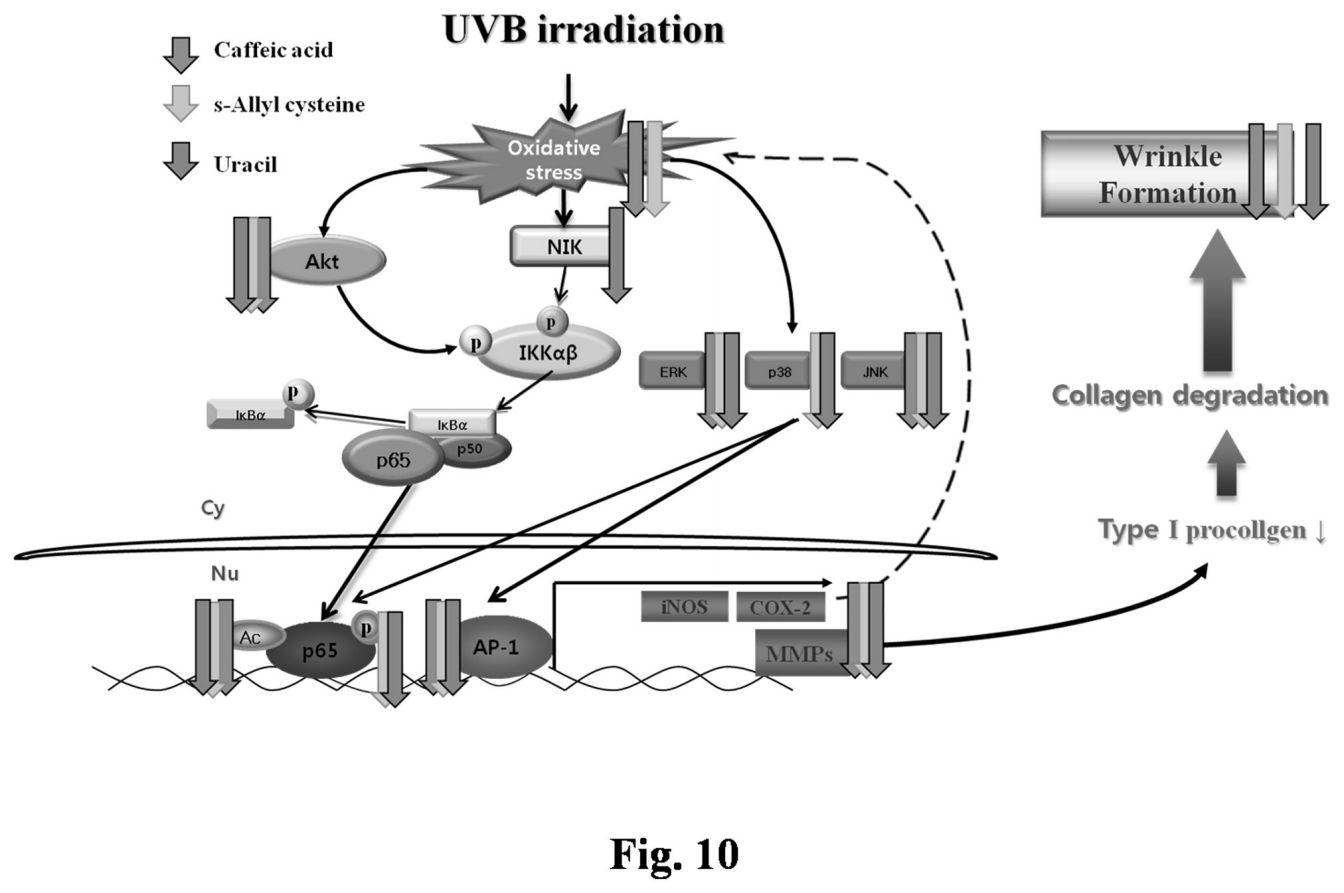
Possible mechanism of active three garlic compounds about effects on anti-wrinkle. COX-2, cyclooxygenase-2; iNOS, inducible nitric oxide synthase; MMP1, matrix metalloproteinase1; MMP3, metalloproteinase3; MMP9, metalloproteinase9; MMP13, matrix metalloproteinase13; UVB, ultra violet B.

Summarizing, the three active garlic compounds effectively inhibited wrinkle formation induced by UVB irradiation, and this inhibition was attributed to increased type I procollagen production via the downregulation of MMPs and upregulation of TIMP4. Wrinkle formation was also reduced by the CA, SAC, and uracil (Figure 1), which inhibited oxidative stress as follows: 1) CA and SAC decreased oxidative stress by directly affecting scavenging and modulating NF-κB or AP-1. 2) CA, SAC, and uracil exhibited anti-inflammatory effects via the suppressions of COX-2 and iNOS. The findings of this study indicate that the active components in garlic have potential as active ingredients for skin treatments against UVB-induced skin damage. Furthermore, the identification of other active compounds of garlic could lead to more effective treatments for proinflammatory and photoaged conditions (Figure 10).

## Materials and Methods

### 4.1: UVB light source

A Crosslinker 800 series (GEX-800, UVP, CA, USA) 6 lamp, 8 watt units was used throughout the study.

### 4.2: Animal study in HR1 hairless mouse

Six-week-old, male, HR-1 hairless mice were obtained from Hoshino Laboratory Animals (Yashino, Saitama, Japan) and housed in a controlled room (23°C±1°C, 55±5% relative humidity, 12 h light/dark cycle) with free access to water and an ad libitum standard laboratory diet. After an acclimation periods (2 weeks), mice were randomly divided into 8 groups of 5 animals. Active compounds from garlic were dissolved in a propylene glycol/ethanol solution (3:7) to concentrations of 5 or 20 µM. These solutions were diluted to 200 µl and topically applied to dorsal skin (3 cm × 3 cm) once daily; propylene glycol/ethanol solution was used as a control. Animals were exposed to UVB generated by a CROSSLINKER (BEX-800, Ultra-Lum Inc., CA, USA).

The animal protocol used in this study was reviewed and approved by the Pusan National University-Institutional Animal Care and Use Committee (PNU-IACUC; Approval Number PNU 2008-0543) with respect to ethical issues and scientific care.

### 4.3: Reagents

CA, SAC, uracil, and all other chemical reagents were purchased from Sigma-Aldrich (St. Louis, MO, USA). Western blotting detection reagents were obtained from Amersham (Bucks, UK). Polyclonal antibodies to MMPs, TIMP4, Type 1 procollagen, p-p65 (Ser536), p65, p50, cFOS, p-cJun, cJun, histone H1, p-IKKαβ (Thr23), pNIK, pERK, pJNK, p-p38, ERK, JNK and p38 were obtained from Santa Cruz Biotechnology (Santa Cruz, CA, USA). Antibodies to Ac-p65 (K310), pAkt, Akt, and pIKKαβ (Ser176/180) were obtained from Cell Signaling Technology (Cell Signaling, Hertfordshire, UK). Polyvinylidenedifluoride (PVDF) membranes were obtained from Millipore Corporation (Bedford, MA, USA). Sterile plastic ware for tissue culture was purchased from SPL Labware (Seoul, Korea). All other materials used were of the highest available commercial grade.

Uracil was separated from methanoic garlic and then purified by Professor Yung Whan Choi of Pusan National University (Korea).

### 4.4: Masson’s trichrome staining

Skins were fixed in 4% paraformaldehyde over-night at room temperature and stained for melanin using a Fontana-Masson staining kit (American Tech Scientific; Lodi, CA, USA). Briefly, sliced skins were stained with ammoniacal silver solution for 60 min at 60°C, incubated in 0.1% gold chloride, and then in 5% sodium thiosulfate.

### 4.5: Measurement of ROS level

A fluorometric assay was used to determine ROS levels. Non-fluorescent DCFDA is oxidized to the highly fluorescent 2', 7'-dichlorofluorescin (DCF) in the presence of esterases and ROS, including lipid peroxides [33]. For cell homogenates, ROS generation was measured using a fluorescence probe, as previously described [34]. Briefly, 50 µM DCFDA was added to homogenates to a final volume of 250 µl. Changes in fluorescence intensity were measured every 5 min for 30 min using a fluorescence plate reader, GENios (Tecan Instruments, Salzburg, Austria) at excitation and emission wavelengths of 485 and 530 nm, respectively.

### 4.6: Western blotting

Western blotting was carried out as described previously [35]. Lysed samples were boiled for 5 min with gel**-**loading buffer (0.125 M Tris-HCl, pH 6.8, 4% SDS, 10% 2-mercaptoethanol and 0.2% bromophenol blue) at a volume ratio of 1:1. Total protein-equivalents were separated by sodium dodecyl sulfate-polyacrylamide gel electrophoresis (SDS-PAGE) using 10% acrylamide gels, as described by Laemmli [36], and transferred to PVDF membranes at 15 V for 1 h in a semi-dry transfer system. Membranes were immediately placed in blocking buffer (5% non-fat milk) in 10 mM Tris (pH 7.5), 100 mM NaCl, and 0.1% Tween 20. Blots was allowed to block at room temperature for 1 h. Membrane were incubated with appropriate specific primary antibodies at 4°C overnight, and then treated with horse radish peroxidase-conjugated anti-mouse antibody (Santa Cruz, 1:10,000), anti-rabbit antibody (Santa Cruz, 1:10,000), or anti-goat antibody (Santa Cruz, 1:10,000) at 25°C for 1 h. Antibody labeling was detected by West-zol Plus and chemiluminescence Fluorchem TMSP (Alpha Innotech Corporation, San Leandro, CA, USA). Pre-stained protein markers were used for molecular weight determinations.

### 4.7: Statistical analysis

For Western blotting, one blot representative of three independent experiments is shown. For other assays, results are expressed as means±SEs. One-factor analysis of variance (ANOVA) followed by Fischer’s protected least significant difference post hoc test were used to determine the significances of group differences. Statistical significance was accepted for p values < 0.05. 

## References

[1] Sjerobabski Masnec I, Poduje S (2008) Photoaging. Coll Antropol 32 Suppl 2: 177-180. PubMed: 19140280. 19140280

[2] Fisher GJ, Kang S, Varani J, Bata-Csorgo Z, Wan Y et al. (2002) Mechanisms of photoaging and chronological skin aging. Arch Dermatol 138: 1462-1470. doi:10.1001/archderm.138.11.1462. PubMed: 12437452. 12437452 10.1001/archderm.138.11.1462

[3] Quan T, Qin Z, Xia W, Shao Y, Voorhees JJ et al. (2009) Matrix-degrading metalloproteinases in photoaging. J Investig Dermatol Symp Proc 14: 20-24. doi:10.1038/jidsymp.2009.8. PubMed: 19675548. 10.1038/jidsymp.2009.8 PMC2909639 19675548

[4] Liu M, Dhanwada KR, Birt DF, Hecht S, Pelling JC (1994) Increase in p53 protein half-life in mouse keratinocytes following UV-B irradiation. Carcinogenesis 15: 1089-1092. doi:10.1093/carcin/15.5.1089. PubMed: 8020138. 10.1093/carcin/15.6.1089 8020138

[5] de Gruijl FR, Sterenborg HJ, Forbes PD, Davies RE, Cole C et al. (1993) Wavelength dependence of skin cancer induction by ultraviolet irradiation of albino hairless mice. Cancer Res 53: 53-60. PubMed: 8416751. 8416751

[6] de Gruijl FR (2000) Photocarcinogenesis: UVA vs UVB. Methods Enzymol 319: 359-366. doi:10.1016/S0076-6879(00)19035-4. PubMed: 10907526. 10907526 10.1016/s0076-6879(00)19035-4

[7] Cooper SJ, Bowden GT (2007) Ultraviolet B regulation of transcription factor families: roles of nuclear factor-kappa B (NF-kappaB) and activator protein-1 (AP-1) in UVB-induced skin carcinogenesis. Curr Cancer Drug Targets 7: 325-334. doi:10.2174/156800907780809714. PubMed: 17979627. 17979627 10.2174/156800907780809714 PMC2605645

[8] Pillai S, Oresajo C, Hayward J (2005) Ultraviolet radiation and skin aging: roles of reactive oxygen species, inflammation and protease activation, and strategies for prevention of inflammation-induced matrix degradation - a review. Int J Cosmet Sci 27: 17-34. doi:10.1111/j.1467-2494.2004.00241.x. PubMed: 18492178. 18492178 10.1111/j.1467-2494.2004.00241.x

[9] Fineschi S, Cozzi F, Burger D, Dayer JM, Meroni PL et al. (2007) Anti-fibroblast antibodies detected by cell-based ELISA in systemic sclerosis enhance the collagenolytic activity and matrix metalloproteinase-1 production in dermal fibroblasts. Rheumatology (Oxf) 46: 1779-1785. doi:10.1093/rheumatology/kem241. PubMed: 17982166. 10.1093/rheumatology/kem241 17982166

[10] Ishitsuka Y, Maniwa F, Koide C, Douzaki N, Kato Y et al. (2007) Detection of modified tyrosines as an inflammation marker in a photo-aged skin model. Photochem Photobiol 83: 698-705. doi:10.1562/2006-07-24-RA-978. PubMed: 17576380. 17576380 10.1562/2006-07-24-RA-978

[11] Ishitsuka Y, Maniwa F, Koide C, Kato Y, Nakamura Y et al. (2012) Increased halogenated tyrosine levels are useful markers of human skin ageing, reflecting proteins denatured by past skin inflammation. Clin Exp Dermatol 37: 252-258. doi:10.1111/j.1365-2230.2011.04215.x. PubMed: 22409522. 22409522 10.1111/j.1365-2230.2011.04215.x

[12] Fisher GJ, Voorhees JJ (1998) Molecular mechanisms of photoaging and its prevention by retinoic acid: ultraviolet irradiation induces MAP kinase signal transduction cascades that induce Ap-1-regulated matrix metalloproteinases that degrade human skin in vivo. J Investig Dermatol Symp Proc 3: 61-68. doi:10.1038/jidsp.1998.15. PubMed: 9732061. 9732061

[13] Azzi A, Gysin R, Kempná P, Munteanu A, Negis Y et al. (2004) Vitamin E mediates cell signaling and regulation of gene expression. Ann N Y Acad Sci 1031: 86-95. doi:10.1196/annals.1331.009. PubMed: 15753136. 15753136 10.1196/annals.1331.009

[14] Pfau JC, Li S, Holland S, Sentissi JJ (2011) Alteration of fibroblast phenotype by asbestos-induced autoantibodies. J Immunotoxicol 8: 159-169. doi:10.3109/1547691X.2011.562257. PubMed: 21457077. 21457077 10.3109/1547691X.2011.562257 PMC3201780

[15] Chiang HM, Lin TJ, Chiu CY, Chang CW, Hsu KC et al. (2011) Coffea arabica extract and its constituents prevent photoaging by suppressing MMPs expression and MAP kinase pathway. Food Chem Toxicol 49: 309-318. doi:10.1016/j.fct.2010.10.034. PubMed: 21056074. 21056074 10.1016/j.fct.2010.10.034

[16] Colin-Gonzalez AL, Santana RA, Silva-Islas CA, Chanez-Cardenas ME, Santamaria A et al. (2012) The antioxidant mechanisms underlying the aged garlic extract- and S-allylcysteine-induced protection. Oxid Med Cell Longev 2012: 907162. 22685624 10.1155/2012/907162 PMC3363007

[17] Rittié L, Fisher GJ (2002) UV-light-induced signal cascades and skin aging. Ageing Res Rev 1: 705-720. doi:10.1016/S1568-1637(02)00024-7. PubMed: 12208239. 12208239 10.1016/s1568-1637(02)00024-7

[18] Yamamoto Y (2001) Role of active oxygen species and antioxidants in photoaging. J Dermatol Sci 27 Suppl 1: S1-S4. doi:10.1016/S0923-1811(01)00091-3. PubMed: 11514118. 11514118 10.1016/s0923-1811(01)00120-7

[19] Beak SM, Lee YS, Kim JA (2004) NADPH oxidase and cyclooxygenase mediate the ultraviolet B-induced generation of reactive oxygen species and activation of nuclear factor-kappaB in HaCaT human keratinocytes. Biochimie 86: 425-429. doi:10.1016/j.biochi.2004.06.010. PubMed: 15308331. 15308331 10.1016/j.biochi.2004.06.010

[20] Thilagar S, Jothi NA, Omar AR, Kamaruddin MY, Ganabadi S (2009) Effect of keratin-gelatin and bFGF-gelatin composite film as a sandwich layer for full-thickness skin mesh graft in experimental dogs. J Biomed Mater Res B Appl Biomater 88: 12-16. PubMed: 18161832. 18161832 10.1002/jbm.b.31024

[21] Lee SM, Lee CT, Kim YW, Han SK, Shim YS et al. (2006) Hypoxia confers protection against apoptosis via PI3K/Akt and ERK pathways in lung cancer cells. Cancer Lett 242: 231-238. doi:10.1016/j.canlet.2005.11.001. PubMed: 16427189. 16427189 10.1016/j.canlet.2005.11.001

[22] Leo L, Leone A, Longo C, Lombardi DA, Raimo F et al. (2008) Antioxidant compounds and antioxidant activity in "early potatoes". J Agric Food Chem 56: 4154-4163. doi:10.1021/jf073322w. PubMed: 18476702. 18476702 10.1021/jf073322w

[23] Kim JM, Lee JC, Chang N, Chun HS, Kim WK (2006) S-Allyl-L-cysteine attenuates cerebral ischemic injury by scavenging peroxynitrite and inhibiting the activity of extracellular signal-regulated kinase. Free Radic Res 40: 827-835. doi:10.1016/j.freeradbiomed.2005.10.034. PubMed: 17015261. 17015261 10.1080/10715760600719540

[24] Bode AM, Dong Z (2003) Mitogen-activated protein kinase activation in UV-induced signal transduction. Sci STKE, 2003: RE2 PubMed: 12554854 10.1126/stke.2003.167.re2 12554854

[25] Breidenbach M, Rein DT, Schöndorf T, Khan KN, Herrmann I et al. (2006) A new targeting approach for breast cancer gene therapy using the heparanase promoter. Cancer Lett 240: 114-122. doi:10.1016/j.canlet.2005.09.007. PubMed: 16271435. 16271435 10.1016/j.canlet.2005.09.007

[26] Schwartz GK (2005) Development of cell cycle active drugs for the treatment of gastrointestinal cancers: a new approach to cancer therapy. J Clin Oncol 23: 4499-4508. doi:10.1200/JCO.2005.18.341. PubMed: 16002840. 16002840 10.1200/JCO.2005.18.341

[27] Pluemsamran T, Onkoksoong T, Panich U (2012) Caffeic acid and ferulic acid inhibit UVA-induced matrix metalloproteinase-1 through regulation of antioxidant defense system in keratinocyte HaCaT cells. Photochem Photobiol 88: 961-968. doi:10.1111/j.1751-1097.2012.01118.x. PubMed: 22360712. 22360712 10.1111/j.1751-1097.2012.01118.x

[28] Studzinski GP, Rathod B, Rao J, Kheir A, Wajchman HJ et al. (1996) Transition to tetraploidy in 1,25-dihydroxyvitamin D3-resistant HL60 cells is preceded by reduced growth factor dependence and constitutive up-regulation of Sp1 and AP-1 transcription factors. Cancer Res 56: 5513-5521. PubMed: 8968109. 8968109

[29] Fisher GJ, Choi HC, Bata-Csorgo Z, Shao Y, Datta S et al. (2001) Ultraviolet irradiation increases matrix metalloproteinase-8 protein in human skin in vivo. J Invest Dermatol 117: 219-226. doi:10.1046/j.0022-202x.2001.01432.x. PubMed: 11511297. 11511297 10.1046/j.0022-202x.2001.01432.x

[30] Okada Y, Naka K, Kawamura K, Matsumoto T, Nakanishi I et al. (1995) Localization of matrix metalloproteinase 9 (92-kilodalton gelatinase/type IV collagenase = gelatinase B) in osteoclasts: implications for bone resorption. Lab Invest 72: 311-322. PubMed: 7898050. 7898050

[31] Chen Z, Seo JY, Kim YK, Lee SR, Kim KH et al. (2005) Heat modulation of tropoelastin, fibrillin-1, and matrix metalloproteinase-12 in human skin in vivo. J Invest Dermatol 124: 70-78. doi:10.1111/j.0022-202X.2004.23550.x. PubMed: 15654955. 15654955 10.1111/j.0022-202X.2004.23550.x

[32] Taddese S, Jung MC, Ihling C, Heinz A, Neubert RH et al. (2010) MMP-12 catalytic domain recognizes and cleaves at multiple sites in human skin collagen type I and type III. Biochim Biophys Acta 1804: 731-739. doi:10.1016/j.bbapap.2009.11.014. PubMed: 19932771. 19932771 10.1016/j.bbapap.2009.11.014

[33] LeBel CP, Ischiropoulos H, Bondy SC (1992) Evaluation of the probe 2',7'-dichlorofluorescin as an indicator of reactive oxygen species formation and oxidative stress. Chem Res Toxicol 5: 227-231. doi:10.1021/tx00026a012. PubMed: 1322737. 1322737 10.1021/tx00026a012

[34] Kim HJ, Jung KJ, Yu BP, Cho CG, Choi JS et al. (2002) Modulation of redox-sensitive transcription factors by calorie restriction during aging. Mech Ageing Dev 123: 1589-1595. doi:10.1016/S0047-6374(02)00094-5. PubMed: 12470896. 12470896 10.1016/s0047-6374(02)00094-5

[35] Habib A, Créminon C, Frobert Y, Grassi J, Pradelles P et al. (1993) Demonstration of an inducible cyclooxygenase in human endothelial cells using antibodies raised against the carboxyl-terminal region of the cyclooxygenase-2. J Biol Chem 268: 23448-23454. PubMed: 8226870. 8226870

[36] Laemmli UK (1970) Cleavage of structural proteins during the assembly of the head of bacteriophage T4. Nature 227: 680-685. doi:10.1038/227680a0. PubMed: 5432063. 5432063 10.1038/227680a0

